# Glucokinase Regulatory Protein as a Putative Target for Gestational Diabetes Mellitus and Related Complications: Evidence From the Mendelian Randomization Study

**DOI:** 10.1111/1753-0407.70056

**Published:** 2025-02-08

**Authors:** Weian Mao, Guiquan Wang, Xiao Wang, Yan Shen, Shuai Yuan, Lin Wang, Haiyan Yang, Yan Li, Kai Chen, Jun Liu, Xi Dong, Yue Zhao, Liangshan Mu

**Affiliations:** ^1^ Reproductive Medicine Center, Zhongshan Hospital Fudan University Shanghai China; ^2^ Department of Obstetrics and Gynecology The Second Affiliated Hospital of Wenzhou Medical University Wenzhou China; ^3^ Department of Reproductive Medicine, Women and Children's Hospital, School of Medicine, Xiamen University, Xiamen, China; Xiamen Key Laboratory of Reproduction and Genetics Xiamen China; ^4^ Department of Obstetrics The First Affiliated Hospital of Wenzhou Medical University Wenzhou China; ^5^ The First School of Medicine Wenzhou Medical University Wenzhou China; ^6^ Unit of Cardiovascular and Nutritional Epidemiology, Institute of Environmental Medicine Karolinska Institutet Stockholm Sweden; ^7^ Reproductive Medicine Center The First Affiliated Hospital of Wenzhou Medical University Wenzhou China; ^8^ Center for Reproductive Medicine, Department of Obstetrics and Gynecology Peking University Third Hospital Beijing China; ^9^ National Clinical Research Center for Obstetrics and Gynecology, Peking University Third Hospital Beijing China; ^10^ Key Laboratory of Assisted Reproduction, Ministry of Education Beijing China; ^11^ Beijing Key Laboratory of Reproductive Endocrinology and Assisted Reproductive Technology Beijing China; ^12^ Nuffield Department of Population Health University of Oxford Oxford UK

**Keywords:** gestational diabetes mellitus, glucokinase regulatory protein, Mendelian randomization

## Abstract

**Background:**

Gestational diabetes mellitus (GDM) is one of the most common complications of pregnancy and is highly associated with adverse perinatal outcomes and long‐term health problems for the mother and offspring. However, there are respective limitations in the pharmacological strategies for the current treatment of GDM. Glucokinase regulatory protein (GCKR) has been associated with GDM in observational studies and animal experiments and thus represents a potential drug target of interest for investigation.

**Methods:**

We applied two‐sample Mendelian randomization (MR) and colocalization analysis using summary‐level data from genome‐wide association studies of GCKR and GDM. Two‐step MR was used to explore the mediating effects of several metabolic factors on the association. We also applied MR to explore the associations of GCKR levels with GDM‐related outcomes. Finally, we performed a phenome‐wide association study (PheWAS) to query the potential effects of altered GCKR levels across multiple health categories.

**Results:**

We found a significant association between elevated GCKR levels and GDM (OR = 3.466, 95% CI = 2.401–5.002, *p* = 3.16 × 10^−11^), also supported by the colocalization analysis ([*P*_coloc_] = 0.997). The estimates were replicated in an independent study (OR = 2.640, 95% CI = 1.983–3.513, *p* = 2.84 × 10^−11^, *P*_coloc_ = 0.983). Elevated GCKR levels were also associated with higher risk of type 2 diabetes (OR = 2.183, 95% CI = 1.846–2.581, *p* = 6.53 × 10^−20^). Two‐step MR suggested that fasting glucose, fasting insulin, and triglycerides partly mediated the causal relationship. PheWAS found that targeting GCKR may improve renal function and glucose homeostasis but cause dyslipidemia and uric acid abnormalities.

**Conclusions:**

This study provided novel evidence that circulating GCKR levels are causally implicated in GDM and related complications, suggesting that it may be a promising target for treatment.

## Graphical abstract

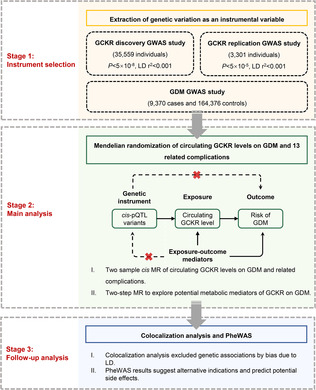

**Summary**

The genetically predicted circulating glucokinase regulatory protein (GCKR) level is causally associated with gestational diabetes mellitus (GDM), and the mediating effects by fasting glucose, triglyceride, and body mass index may provide insights into the underlying mechanisms. The intervention of the target is effective in the management of GDM and related complications; thus, GCKR serves as a potential drug target for further development.

## 1 Introduction

Gestational diabetes mellitus (GDM) is one of the most common complications during pregnancy with a prevalence of 1.8%–31% globally [1]. It is also associated with elevated risks of adverse perinatal outcomes and long‐term health issues for maternal and offspring [2], which bring a heavy financial burden for both individuals and public healthcare systems [3]. Therefore, women with GDM need to be properly treated. Despite lifestyle and dietary interventions, pharmacologic intervention is required for optimal glycemic control when the blood glucose level exceeds the recommended thresholds [4]. Insulin has been considered the standard therapy for GDM that does not cross the placenta; however, it requires multiple daily injections and intensive glucose monitoring to prevent maternal hypoglycemic episodes [4]. Oral glucose‐lowering drugs, such as metformin and glyburide, offer greater convenience but cross the placental barrier, raising concerns about long‐term safety for mothers and offspring [5]. Therefore, alternative therapeutic strategies and novel targets for GDM treatment are warranted.

One promising candidate is the glucokinase regulatory protein (GCKR, also known as GKRP), a 68 kDa polypeptide encoded by the *GCKR* gene; it functions as a competitive inhibitor of glucose binding to glucokinase (GCK), a key regulator of glucose metabolism [6]. The GCK–GCKR maintains glucose homeostasis by regulating hepatic glucose metabolism and insulin release. Observational studies revealed the associations between single nucleotide polymorphisms (SNPs) of *GCKR* and GDM risk [7]. In animal studies, a novel pocket on GCKR has been identified, which can induce GCK protein translocation, resulting in glucose‐lowering effects and thus suggesting GCKR as a potential therapeutic target for GDM [8]. However, the high attrition associated with drug development has prompted further investigation of relevant targets before clinical trials, while genetically supported targets are considered to have a higher success rate [9].

Mendelian randomization (MR), a widely used approach in genetic epidemiology, utilizes analytical estimation based on the exposure‐related genetic variants as instrumental variables (IVs) to ascertain the causal effects of specific exposures on outcomes while minimizing confounding and reverse causality [10]. Using genetic variants located within or near genes encoding drug targets, MR estimates downstream effects similar to the desired drug response to explore the impact on clinical outcomes. Due to its cost‐effectiveness and extensive applications, MR is increasingly used to reveal the genetic basis of proteomics in the early screening of potential therapeutic targets for disease [11].

In this study, we aimed to obtain causal estimates of the relationship between circulating GCKR levels and GDM and mediating effects while assessing the impacts of GCKR on GDM‐related complications to comprehensively evaluate the genetic evidence for GCKR as a therapeutic target. We also discuss the possible indications and side effects of targeting GCKR as a complement for drug target development.

## 2 Methods

### 2.1 Study Design

**FIGURE 1:**
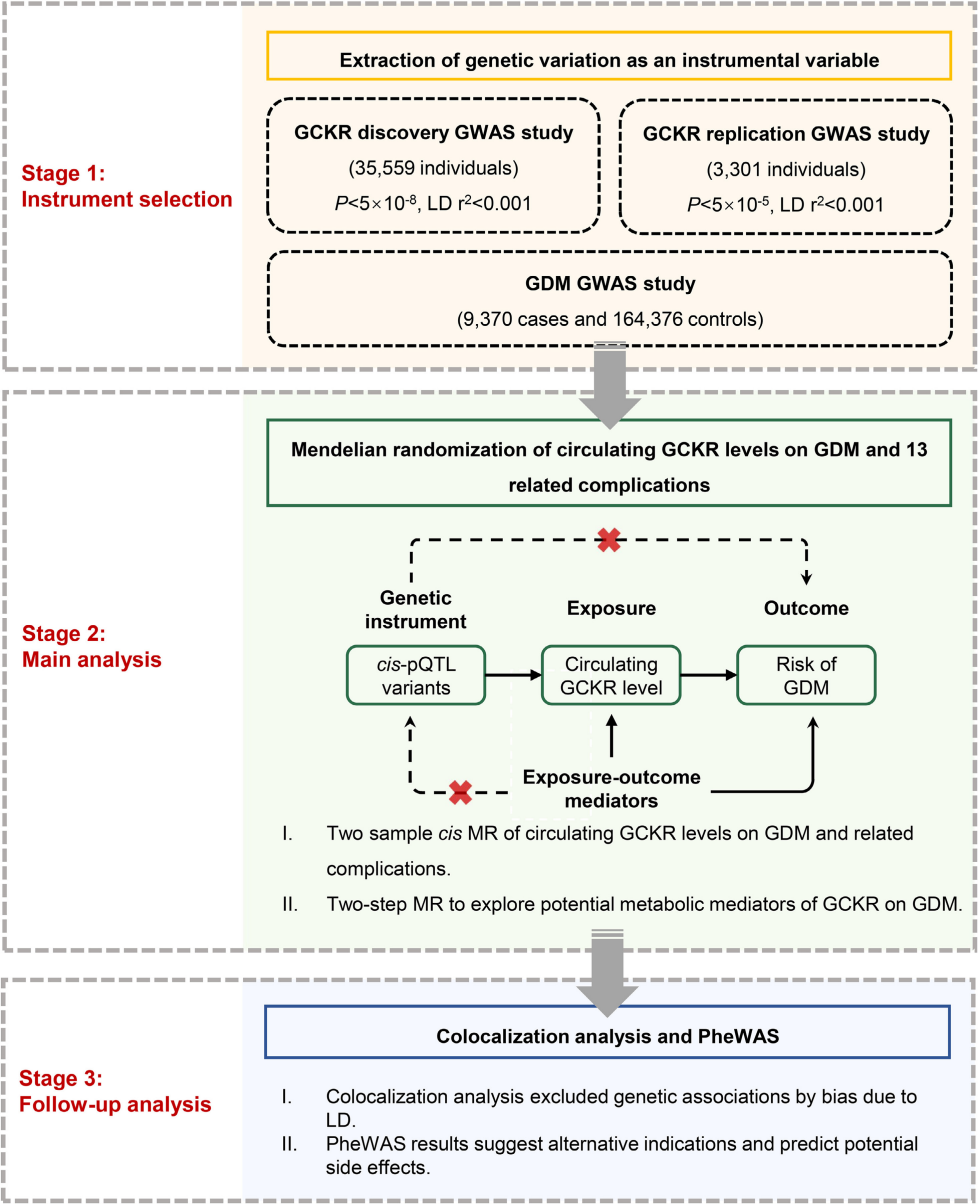
Overview of the study design in our study.

This study followed the Strengthening the Reporting of Observational Studies in Epidemiology Using Mendelian Randomization (STROBE‐MR) guideline (Supplementary Table S1) [12]. The study design overview is illustrated in Figure 1.

We first performed two‐sample univariable MR by using publicly available genome‐wide association study (GWAS) to evaluate the causal effect of genetically predicted circulating GCKR levels on the risk of GDM and related complications. We then explored potential intermediate traits in the pathways from GCKR to GDM and estimated the mediating proportion of identified mediators. We also used a colocalization approach to test the shared causal signals in genetic association between GCKR and GDM. Lastly, we performed a phenome‐wide association study (PheWAS) to query the potential effects of altered GCKR activity in the early stages of drug discovery.

### 2.2 GWASs Involved in the Current Study

We extracted GCKR GWAS data from the largest plasma proteomic study by Ferkingstad et al., including 35 559 Icelandic individuals from the Icelandic Cancer Project and deCODE genetics [13]. We validated the results using GCKR IVs from another independent proteomics study by Sun et al. that included 3301 INTERVAL healthy blood donors in the United Kingdom [14]. Both GWAS datasets were quantified using the Aptamer‐based (SOMAmer) technology. The genetic associations were adjusted for age, sex, duration between blood draw and processing, and specific covariates.

GDM was defined in the FinnGen consortium according to the genotype data from the Finnish Biobank and digital health records (ICD‐9 or ICD‐10 diagnosis, Supplementary Table S2), which included 9370 GDM cases and 164 376 controls [15]. The genetic associations were adjusted for age, sex, and top principal components. We additionally obtained genetic associations for 13 GDM‐related pregnancy or perinatal outcomes, including sporadic miscarriage, multiple consecutive miscarriage, premature rupture of membranes, premature separation of placenta, preterm labor and delivery, eclampsia, pre‐eclampsia, postpartum hemorrhage, postpartum depression, polyhydramnios, gestational hypertension, primary hypertension, and type 2 diabetes (T2DM), from other publicly available GWAS [16]. Genetic association estimates for possible intermediate traits (i.e., fasting glucose, fasting insulin, triglycerides, and body mass index [BMI]) were derived from large‐scale corresponding GWASs [17, 18, 19]. More details on used GWAS in our MR analysis are described in Supplementary Table S2.

### 2.3 Genetic Instrument Selection

MR relies on three core assumptions [10]. First, the genetic variant should be robustly associated with the exposure. The second MR assumption is that the genetic variant should be independent of confounders of the exposure–outcome association. We ensured that genetic associations were derived solely from homogeneous populations of European ancestry to minimize confounding due to ancestral background. The third assumption is that the genetic variant should influence the outcome only via the exposure pathway.

The *cis*‐acting protein quantitative locus (*cis*‐pQTL) was selected as an IV for GCKR levels, which is considered to have higher biological priority for a direct and definite influence on protein abundance. The *GCKR* gene is located at human 2p23.3, and we defined *cis*‐pQTLs as genome‐wide significant SNPs within 1 Mb of the gene encoding region. Conditionally uncorrelated variants at genome‐wide significance (*p* < 5 × 10^−8^, linkage disequilibrium [LD] *r* ^2^ < 0.001) associated with GCKR concentrations were selected as IVs from the study by Ferkingstad et al. [13] The selection was replicated from the study by Sun et al., which passed the threshold of *p* < 5 × 10^−5^ and LD *r* ^2^ < 0.001 due to its smaller sample size [14].

### 2.4 Two‐Sample MR

To estimate the effect of *cis*‐pQTL proxied circulating GCKR levels on the risk of GDM and related complications, we used the Wald ratio method. For binary outcomes, we presented MR estimates in terms of odds ratios (ORs) with 95% confidence intervals (CIs), and for continuous outcomes, we used beta coefficients with standard errors (SEs) per genetically predicted 1‐SD change in circulating GCKR levels. We report two‐sided *p*‐values for the MR results, with a Bonferroni correction for multiple testing threshold (*p* ≤ 3.5 × 10^−3^), as 14 disease traits were considered.

In the reverse MR, we performed two‐sample MR analysis with GDM as exposure and circulating GCKR levels from two studies as outcome. Genetic IVs for GDM passed the threshold of *p* < 5 × 10^−8^ and LD *r* ^2^ < 0.001. The conventional inverse variance weighted (IVW) method was mainly applied, which combines the Wald ratio estimates for multiple genetic variants for the same exposure using the random effects [20]. Additional methods, including MR‐Egger, weighted median, simple mode, and weighted mode, were used as robust sensitivity analyses to produce relatively valid estimates under different assumptions [21].

### 2.5 Two‐Step MR for Mediation Analysis

We further assessed direct effects (i.e., the effect of circulating GCKR levels on GDM independent of mediators) and indirect effects (i.e., the effect of circulating GCKR levels on GDM through mediators only) using two‐step multivariate MR (MVMR) [22]. In the first step, the mediators (i.e., fasting glucose, fasting insulin, triglycerides, and BMI) were regressed on GCKR. We performed a series of two‐sample MR analyses to assess the associations of GCKR with intermediate traits. In the second step, MVMR was used subsequently to further identify the effects of potential mediators by using combined IVs for GCKR and intermediate traits. We calculated the mediating effect by the coefficient product method and also estimated the proportion of the mediating effect and the estimated SE.

All MR analyses were performed with the package “TwoSampleMR” implemented in R software version 4.0.5 [16].

### 2.6 Power Calculation

We calculated *F*‐statistics by the formula *F* = (*N* − *K* − 1/*K*)(*R* ^2^/1 − *R* ^2^), where *R* ^2^ indicates the proportion of variance in the phenotype explained by the instrument, *N* is the sample size, and *K* is the number of instruments [23]. *F*‐Statistics estimate the bias from weak instruments depending on the strength of the instrument with a threshold of 10 as the conventional rule. *R* ^2^ indicates the proportion of variance in the phenotype explained by the instrument as 2EAF(1 − EAF)beta^2^, where EAF is the effect allele frequency and beta is the effect size of the effect allele of the IVs.

### 2.7 Colocalization Analysis

We used a Bayesian statistical method for the two plasma protein studies and the GDM study to test colocalization of a shared genetic signal between circulating GCKR levels and GDM and thus to distinguish whether two traits have a common association signal or independent signals associated with separate traits [24]. The prior probabilities for the genetic signal were set with default values, P1 = P2 = 1 × 10^−4^ and P12 = 1 × 10^−5^, where P1 and P2 are the probabilities that a given genetic signal is associated with either of the two traits, and P12 is the probability associated with both traits [24]. It computes the posterior probability (PP) of association for five alternative hypotheses: H0, no association with either trait; H1, signal unique to the circulating GCKR; H2, signal unique to GDM; H3, two distinct causal variants in the same locus, one for each trait; and H4, presence of a shared causal variant between circulating GCKR and GDM. PPH4 values of 80% or higher were considered evidence of colocalization. The SNP.PP.H4 presents the PP that a specified SNP supports causality as a shared signal, which only makes sense if the posterior support for H4 is convincing. We used *p*‐values and minor allele frequency obtained from the SNPs in the *GCKR* encoded region ±200 kb to perform colocalization using the R package “coloc” [24].

### 2.8 Phenome‐Wide Association Study

To further detect the potential pleiotropic effect of GCKR *cis*‐pQTLs, we applied PheWAS by using 3302 unique traits from multiple domains in the publicly available GWAS ATLAS database [25]. The integration of MR and PheWAS scans a comprehensive range of all measured phenotypes, which can be used to appraise the potential role of GCKR in multiple relationships simultaneously. Since the investigated phenotypes were not completely independent, the Benjamini–Hochberg false discovery rate (FDR) correction was applied to analyze with a threshold of 5% for multiple testing.

## 3 Results

### 3.1 Genetic Instruments for GCKR

Two pQTLs associated with circulating GCKR levels were used as instruments: *cis*‐pQTL (rs1260326 within the *GCKR*) from the study by Ferkingstad et al. and *cis*‐pQTL (rs780094 within the *GCKR*) from Sun et al.'s study. The *F*‐statistic for *cis*‐pQTL of Ferkingstad et al.'s study was 107.0, with 0.3% of the variation in GCKR. In the replication study of Sun et al., the *F*‐statistic for *cis*‐pQTL was 19.3, with 0.6% of the variation in circulating GCKR level (Table 1).

**TABLE 1:** Mendelian randomization estimates of assessing the causal association between circulating GCKR and GDM.

Exposure	Outcome	Study source	Method	*N*	MR β (SE)	*p*	OR	MR 95% CI	*R* ^2^	*F*‐Statistics
GCKR	GDM	Ferkingstad et al.	Wald ratio	1	1.24 (0.187)	3.16 × 10^–11^ *	3.466	(2.401, 5.002)	0.003	107.0
GCKR	GDM	Sun et al.	Wald ratio	1	0.97 (0.146)	2.84 × 10^–11^ *	2.640	(1.983, 3.513)	0.006	19.3
GDM	GCKR	Ferkingstad et al.	IVW	4	0.15 (0.375)	0.69	1.160	(0.557, 2.416)	0.013	572.1
GDM	GCKR	Sun et al.	IVW	9	0.13 (0.088)	0.13	1.141	(0.960, 1.355)	0.005	97.0

Abbreviations: GCKR, glucokinase regulatory protein; GDM, gestational diabetes; IVW, inverse‐variance weighted; MR, Mendelian randomization; *N*, number of SNPs; OR, odds ratio; SE, standard error.

* Significance at *p* < 0.05.

### 3.2 Association of GCKR With GDM Risk and Related Complications

Genetically predicted higher levels of GCKR were associated with a higher risk of GDM (OR per SD = 3.466, 95% CI = 2.401–5.002, *p* = 3.16 × 10^−11^). The association was replicated in the analysis using a genetic instrument from the Sun et al. study (OR per SD = 2.640, 95% CI = 1.983–3.513, *p* = 2.84 × 10^−11^). As for reverse MR analysis, there was no association between GDM and circulating GCKR levels in either Ferkingstad et al. GWAS (beta = 0.144, *p* = 0.25) or Sun et al. GWAS (beta = 0.132, *p* = 0.13).

**FIGURE 2:**
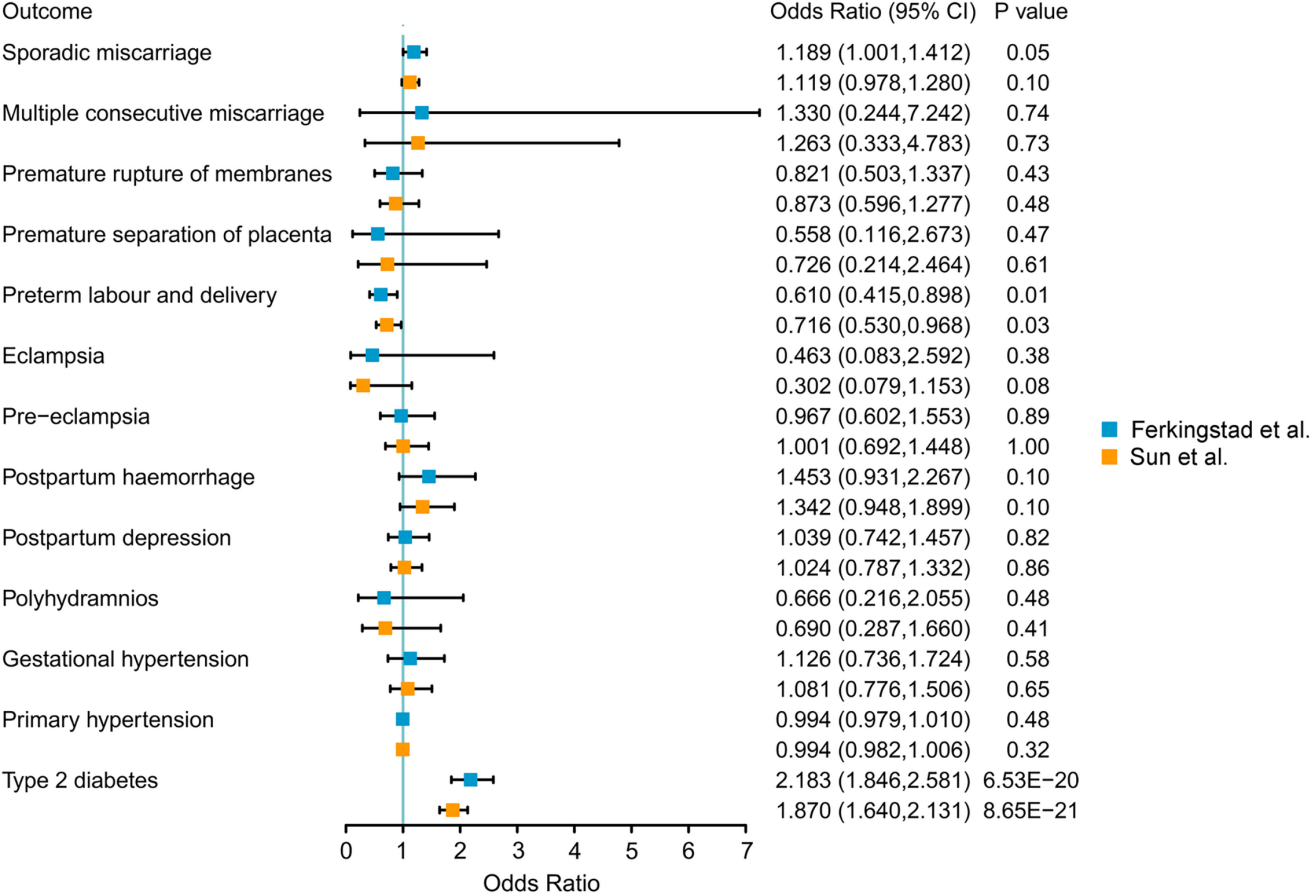
Associations between genetically predicted GCKR and GDM‐related complications. The forest plot shows Mendelian randomization effect estimates and 95% confidence intervals for the genetic proxied effect of GCKR and outcomes.

Using Bonferroni‐corrected *p*‐value ≤ 3.5 × 10^−3^ as the significance threshold after correction for multiple testing, genetically predicted higher levels of circulating GCKR were associated with the increased risk of T2DM (OR = 2.183, 95% CI = 1.846–2.581, *p* = 6.53 × 10^−20^). These associations were observed in the replication analysis for T2DM (OR = 1.870, 95% CI = 1.640–2.131, *p* = 8.65 × 10^−21^) (Figure 2 and Supplementary Table S3).

### 3.3 Mediation MR Analysis

We applied MR‐based mediation analysis to study the role of intermediate metabolic factors in mediating the relationships between circulating GCKR level and GDM. The effect of circulating GCKR on GDM was attenuated when correcting for only one of the following mediators: fasting glucose, fasting insulin, triglycerides, and BMI (Supplementary Table S4). Even if these metabolic mediators were all adjusted, the effect of GCKR on GDM still remained statistically significant (OR = 1.329, 95% CI = 1.037–1.702, *p* = 2.46 × 10^−2^).

Additionally, fasting glucose (beta ± SE = 2.519 ± 0.248, *p* = 2.58 × 10^−24^), triglycerides (beta ± SE = 0.163 ± 0.050, *p* = 1.08 × 10^−3^), and BMI (beta ± SE = 0.522 ± 0.067, *p* = 9.65 × 10^−15^) were all positively correlated with the risk of GDM after adjustment for circulating GCKR, indicating that the associations among these metabolic traits and the risk of GDM were independent of GCKR (Table 2). We further calculated the proportion of the circulating GCKR effect mediated by fasting glucose, triglycerides, and BMI for GDM; in this way, the effects of GCKR and mediators on GDM outcomes were significant at unadjusted *p* < 0.05. We found that fasting glucose (66.07% of the effect of GCKR on GDM), triglycerides (15.63% of the effect of GCKR on GDM), and BMI (5.08% of the effect of GCKR on GDM) each mediated the total genetically predicted effects of the protein trait on GDM partially (Table 2).

**TABLE 2:** Proportion of effect mediated for exposure–mediator–outcome relationships.

Exposure	Mediator	Outcome	Log OR (SE) per 1SD higher exposure, *p*‐value			Proportion of effect mediated (95% CI)
			Exposure‐outcome	Mediator‐outcome	Exposure‐mediator	
GCKR	Fasting glucose	GDM	1.243 (0.187)	2.519 (0.248)	0.326 (0.020)	66.07%
			*P* = 3.16 × 10^−11^	*P* = 2.58 × 10^−24^	*P* = 8.48 × 10^−62^	(41.47%–90.66%)
	Triglycerides			0.163 (0.050)	−1.192 (0.023)	15.63%
				*P* = 1.08 × 10^−03^	*P* < 5.00 × 10^−300^	(5.15%–26.12%)
	BMI			0.522 (0.067)	0.121 (0.020)	5.08%
				*P* = 9.65 × 10^−15^	*P* = 6.56 × 10^−10^	(2.51%–7.65%)
	Fasting insulin			0.777 (0.438)	0.267 (0.022)	NA
				*P* = 7.61 × 10^−02^	*P* = 5.21 × 10^−34^	NA

Abbreviations: BMI, body mass index; CI, confidence interval; GCKR, glucokinase regulatory protein; GDM, gestational diabetes; NA, not applicable; OR, odds ratio; SD, standard deviation; SE, standard error.

### 3.4 Colocalization Analysis

**FIGURE 3:**
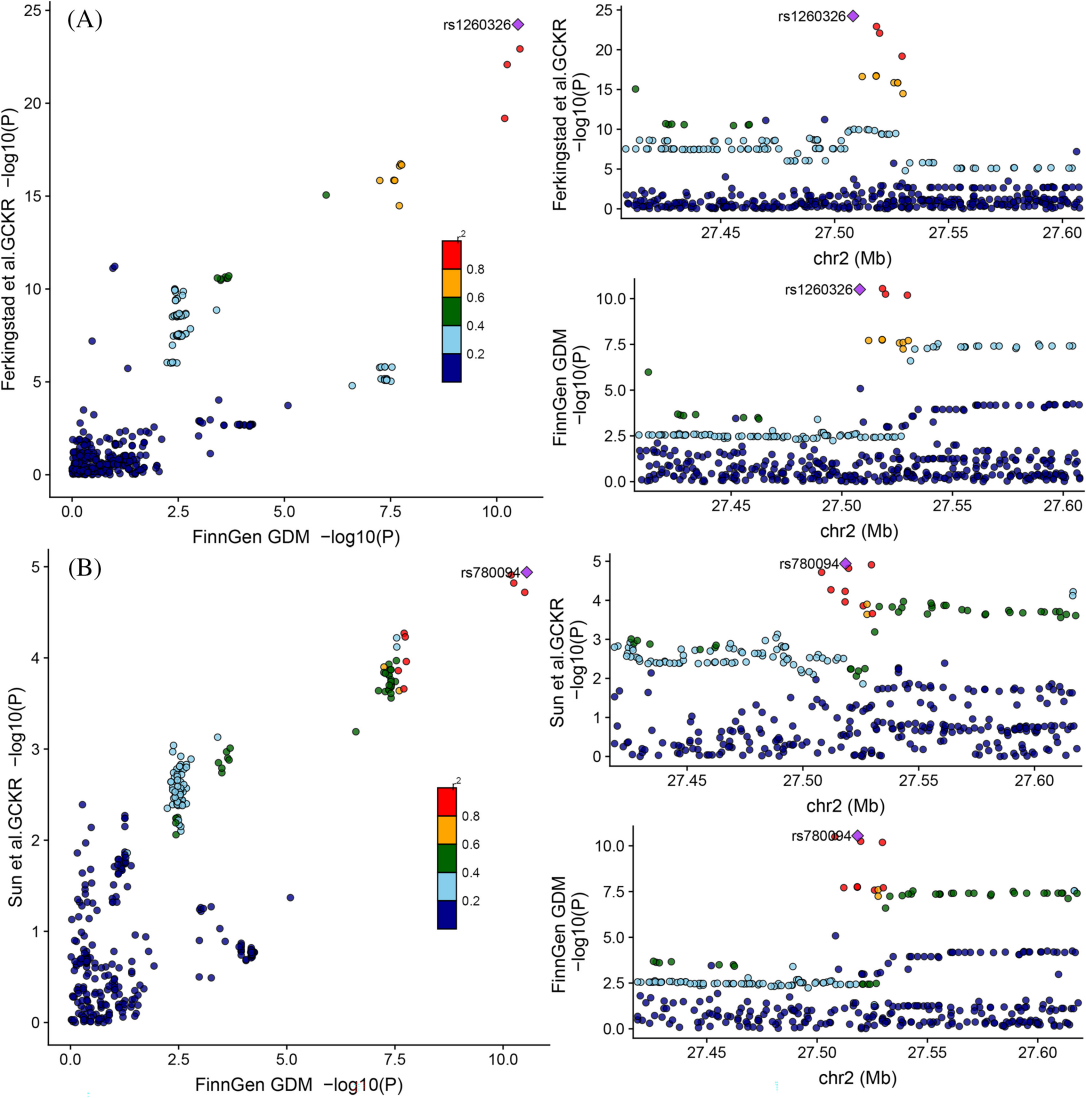
Regional Manhattan plot of associations of SNPs with GCKR and GDM locus. (A) LocusCompare plots comparing genetic signals at ±200 kb of GCKR for circulating GCKR levels (Ferkingstad et al.) and GDM. (B) LocusCompare plots comparing genetic signals at ±200 kb of GCKR for circulating GCKR levels (Sun et al.) and GDM. The plots show the lead SNP with the highest posterior probability according to “coloc”.

To assess confounding due to LD, we tested the probability whether the genetic determinants of the GCKR level were shared with those of GDM using colocalization. The colocalization results support the causal association between GCKR and GDM by strong evidence of colocalization (PP_H4_ = 0.997), indicating that GCKR and GDM were linked through the shared variant in the 200 kb locus around the *GCKR*, and the *cis*‐pQTL rs1260326 is likely to alter circulating GCKR levels to affect GDM (SNP. PPH4 = 0.89) (Figure 3A and Supplementary Tables S5 and S6). Similarly, there was a significant chance (PP_H4_ = 0.983) that the GCKR level and GDM shared a common genetic signal (PP_H4_ = 0.983) in the replication analysis (Figure 3B and Supplementary Tables S5 and S7). These results suggest that there are single shared causal signals in the same locus, distinguishing causal effects from confounding by excluding LD bias.

### 3.5 PheWAS Results

The robust *cis*‐pQTLs of circulating GCKR were significantly (FDR < 0.05) and extensively associated with traits from the PheWAS database. The most highly related traits were in the metabolic domain, which are triglycerides, weight maintenance, and fasting glucose effects. The second highly related trait was alcohol intake frequency in the psychiatric domain. Other highly related traits include cholesterol, diabetes, glomerular filtration rate, serum urate, etc. (Supplementary Figures S1 and S2). It is assumed that using GCKR as a therapeutic target for GDM reduces the plasma glucose level and also improves the glomerular filtration rate while, on the other hand, leading to hypertriglyceridemia and gout. As such, resultant associations can be interpreted as concomitant side effects expected to arise if GCKR is targeted therapeutically.

## 4 Discussion

In this study, we found that the elevated circulating GCKR level was causally associated with the risk of GDM, which was supported by strong evidence of colocalization. The MR analysis for perinatal outcomes and complications explored the effects of circulating GCKR interventions on GDM‐related adverse health outcome estimates. Two‐step MR showed that the causality between circulating GCKR and GDM was mediated by fasting glucose, triglyceride, and BMI. PheWAS further predicted that the intervention of GCKR can simultaneously prevent kidney disease but may be susceptible to side effects (e.g., dyslipidemia, gout), which need further research. Taken together, our results supported the hypothesis that circulating GCKR may serve as a potential therapeutic target for GDM.

The relationship between GCKR and GDM involves several complex metabolic mechanisms, mainly focusing on glucose metabolism, insulin sensitivity, and lipid metabolism. GCKR is one of the most important physiological inhibitors of GCK and plays a critical role in glucose homeostasis. GCKR sequesters the metabolic enzyme in the nucleus of hepatocytes and modulates insulin secretion in the pancreas [26]. The dissociation of the GCK–GCKR complex in the liver promotes the translocation of GCK to the cytoplasm, thereby increasing hepatic glucose phosphorylation, insulin release, and glycogen synthesis [27]. It should be noted that if it overloads the liver's ability to process glucose to synthesize glycogen, it stimulates the de novo production of lipids. Enhanced insulin resistance is a normal physiological phenomenon during pregnancy, which is closely linked to a range of hormones, metabolic demands, and physiological changes, aiming at adapting to the energy demands of fetal growth and development. However, if this resistance is excessive or cannot be effectively regulated, it may lead to GDM. On the background of enhanced insulin resistance underlying pregnancy, higher circulating GCKR levels may contribute to GDM during pregnancy by inhibiting GCK activity, reducing hepatic clearance of glucose, and also impairing glucose perception by pancreatic β‐cells, further exacerbating the risk of insulin resistance and hyperglycemia [28].

In addition, valuable insights can be learned from humans carrying *GCKR* gene variants, as these loci have biological effects similar to exogenous disruptors. Integrating genetic and metabolomic data, Liu et al. reported that maternal carrying mutations inducing high GCKR expression levels have lower levels of palmitoleic acid and that serum palmitoleic acid levels are negatively correlated with insulin sensitivity, demonstrating a potential role for GCKR‐regulated metabolites in pregnancy‐induced insulin resistance [26]. Recent studies also reported further correlations between GCKR and maternal fasting and 1‐h metabolites that are widely associated with specific GDM subtypes (insulin‐resistant GDM and insulin‐deficient GDM) [29]. Specifically, *GCKR*‐mediated branched‐chain amino acids were associated with insulin‐resistant GDM, and multiple acylcarnitines were associated with insulin‐deficient GDM; only two metabolites were oriented in the same direction in the two subtypes, suggesting a mechanism by which the GCKR‐regulated distinct metabolome is involved in the development of different subtypes of GDM [29]. However, evidence is scarce from our mediation analysis to determine that fasting insulin drives the causal relationship between circulating GCKR and GDM. As found by Perez‐Martinez et al., interaction between rs1260326 and plasma Omega‐3 polyunsaturated fatty acids triggered insulin resistance; thus, the role of fasting insulin in GCKR‐driven GDM may be explained partially by gene–fatty acid interactions [30]. Regulation of GCKR may lead to altered the status of pre‐pregnancy overweight, glucose, and triglycerides, which have proven to play an important role in the development and progression of GDM [31]. Therefore, GCKR affects the regulation of glycolysis and lipid metabolism through alterations in the above pathways, which may contribute to GDM occurrence.

As GCKR represents a potential therapeutic strategy or drug target for GDM, it is also of great interest to conduct targeted drug development. Although no drugs have been developed and clinical trials conducted for GDM using GCKR as a target, several existing lines of evidence suggest that improvements in insulin resistance and metabolic control with GCKR‐targeted interventions may exert a similar effect on the control of GDM. Two optimized molecules, AMG‐1694 and AMG‐3969, were advanced into preclinical models of diabetes and demonstrated efficacy in all tested diabetic models by reversing the inhibitory effect of GCKR on GCK activity and promoting GCK translocation [32]. The glucose‐lowering effects induced by these GCKR small‐molecule disruptors was restricted to diabetic and non‐normoglycaemic animals, suggesting an advantage in preventing adverse effects of drug treatment, which may lead to hypoglycemia. In addition, as a regulatory target of GCKR, GCK has been developed as an effective and clinically applicable oral hypoglycemic agent (e.g., Dorzagliatin), which implies the feasibility of targeting GCKR for the development of drugs for GDM [33, 34].

The primary aims of the treatment of GDM are to prevent maternal and fetal complications [35]. MR analysis of GDM‐related suggested that GCKR as an intervention target for GDM treatment appears to achieve this aim, including lower rates of T2DM. Although hyperglycemic symptoms in patients with GDM generally resolve postpartum, there appears to be consensus that women with a history of GDM appear to have a significantly higher risk of developing T2DM than those with normal glucose levels during pregnancy [36]. In addition, fetal macrosomia characterized by fetal weight increase is another common adverse outcome of GDM. For the mother, it can lead to cesarean section, postpartum hemorrhage, and vaginal laceration, while for the infant, macrosomia increases the risk of complications such as obstructed shoulder births, preterm delivery, and brachial plexus injury [37]. These results provide complementary evidence of the clinical implication of GCKR as a therapeutic target to optimize maternal and neonatal outcomes.

Since the high failure rate in clinical trials for new drug development can be attributable to unintended drug effects in most cases, PheWAS validates that disease associations in disease diagnostic cohorts with extensive health information more closely resemble that in the real world and the research findings enrich the drug discovery process. Our PheWAS results provided a glimpse of the expected beneficial and detrimental effects of GCKR disruptors as a potential new class of hypoglycemic drugs, which are critical if someday they may be used in clinical practice. As these *cis*‐pQTLs are considered to be functional SNPs accounting for the association of *GCKR* loci with multiple traits, their association with traits in multiple domains needs to be examined. The effect allele of *GCKR cis*‐pQTLs was always associated with higher glucose, insulin resistance, obesity, and other traits; meanwhile, it reduced serum uric acid, alcohol consumption, and blood lipids, which implies a side effect of GCKR intervention. As previously suggested, despite the advantages of modulating GCKR level in improving the regulation of glucose metabolism and reducing the risk of diabetes and kidney disease, its potential adverse side effects such as gout and dyslipidemia need to be taken into account [38]. Observational studies have shown that the glucose‐lowering and triglyceride‐raising allele does not elevate the cardiovascular risk [39], thus providing safety proof for GCKR as a target for pharmacological intervention. Overall, the modulation of the GCK–GCKR complex appears to be a double‐edged sword that requires a combination of desired therapeutic efficacy and tolerance of unfavorable side effects. Future efforts regarding GCKR target development should focus on other forms of diabetes (either genetic or T2DM in general) and related metabolic diseases such as metabolic dysfunction‐associated fatty liver disease [40].

To the best of our knowledge, our study is the first to explore the causal relationship between circulating GCKR level and GDM risk. The main strength of our study is the comprehensive, multi‐step research evidence that demonstrates the causal relationship between circulating GCKR levels and GDM and the significance of potential drug target development in details.

There are several limitations to this study. First, considering the multiple effects of GCKR genetic variants on metabolism, it is difficult to exclude the potential effect of directional pleiotropy completely, and this relevance should be interpreted with caution. Second, the inclusion of cases of GDM from the FinnGen study was confirmed from digital health record data, which may lead to a slight imprecision in the definition of the disease outcome. Third, our analysis was restricted only to European ancestry. Due to differences in demographic relationships, allele frequencies, and local LD patterns, whether our findings can be generalized to other ethnicities needs to be verified in future studies including multi‐ethnic cohorts [41]. Fourth, MR results reflect the lifetime effects of a target, which is not equivalent to an intervention at a specific point in time or over a shorter period of time.

In conclusion, we provide novel evidence that circulating level of GCKR is causally implicated in the risk of GDM and related complications, suggesting that it may be a promising target for GDM. Further studies are warranted to elucidate the metabolic pathways and translational implications of mediators involved in the relevant association of circulating GCKR with GDM. Meanwhile, large‐scale randomized controlled trials are also warranted to further validate our findings of a protective effect of GCKR as a therapeutic target against GDM. For target development, future work should consider more of the impact on other forms of diabetes and related metabolic diseases.

## Author Contributions

L.S.M. and Y.Z. designed the study. W.A.M., J.L., G.Q.W., and Y.S. performed the MR analyses. W.A.M., G.Q.W., X.W., and K.C. drafted the manuscript. X.D., S.Y., L.W., H.Y.Y., and Y.L. revised the manuscript.

## Disclosure

The authors declare that they have no known competing financial interests or personal relationships that could have appeared to influence the work reported in this paper.

## Ethics Statement

The ethical approvals and consent to participate involved in the MR section were available in the original GWAS study.

## Conflicts of Interest

The authors declare no conflicts of interest.

## Supporting information


**Supplementary Table 1.** The STROBE‐MR‐checklist applicable to the current study.
**Supplementary Table 2**. Characteristics of the GWAS included in Mendelian randomization analysis.
**Supplementary Table 3**. The effect of GCKR instrumented by *cis*‐pQTL on GDM‐related complications.
**Supplementary Table 4**. Multivariable Mendelian randomization effect estimates of GCKR on GDM, adjusted for metabolic mediators.
**Supplementary Table 5**. Results of colocalization analysis for comparisons showing evidence for colocalization (H4) in the main analysis.
**Supplementary Table 6**. Results of top 10 SNPs with highest probability in colocalization analysis of GCKR (Ferkingstad et al.) and GDM.
**Supplementary Table 7**. Results of top 10 SNPs with highest probability in colocalization analysis of GCKR (Sun et al.) and GDM.
**Supplementary Figure 1**. Results of the phenome‐wide association study of *cis*‐pQTL for GCKR (Ferkingstad et al.).
**Supplementary Figure 2**. Results of the phenome‐wide association study of *cis*‐pQTL for GCKR (Sun et al.).

## Data Availability

All data generated or analyzed during this study are included in the main text and its supplementary information files.
