# Supplementary material for: Glucokinase Regulatory Protein as a Putative Target for Gestational Diabetes Mellitus and Related Complications: Evidence From the Mendelian Randomization Study
Source: J Diabetes. 2025 Feb 8;17(2):e70056. doi: 10.1111/1753-0407.70056 (PMC11806411; doi:10.1111/1753-0407.70056)
Supplement: Supplementary file 1 — Supplementary Table 1. The STROBE‐MR‐checklist applicable to the current study. Supplementary Table 2. Characteristics of the GWAS included in Mendelian randomization analysis. Supplementary Table 3. The effect of GCKR instrumented by cis‐pQTL on GDM‐related complications. Supplementary Table 4. Multivariable Mendelian randomization effect estimates of GCKR on GDM, adjusted for metabolic mediators. Supplementary Table 5. Results of colocalization analysis for comparisons showing evidence for colocalization (H4) in the main analysis. Supplementary Table 6. Results of top 10 SNPs with highest probability in colocalization analysis of GCKR (Ferkingstad et al.) and GDM. Supplementary Table 7. Results of top 10 SNPs with highest probability in colocalization analysis of GCKR (Sun et al.) and GDM. Supplementary Figure 1. Results of the phenome‐wide association study of cis‐pQTL for GCKR (Ferkingstad et al.). Supplementary Figure 2. Results of the phenome‐wide association study of cis‐pQTL for GCKR (Sun et al.). [file JDB-17-e70056-s001.docx]

**Supplemental Content**

**Supplementary Table 1.** The STROBE-MR-checklist applicable to the current study

**Supplementary Table 2.** Characteristics of the GWAS included in Mendelian randomization analysis

**Supplementary Table 3.** The effect of GCKR instrumented by *cis*-pQTL on GDM related complications

**Supplementary Table 4.** Multivariable Mendelian randomization effect estimates of GCKR on GDM, adjusted for metabolic mediators

**Supplementary Table 5.** Results of colocalization analysis for comparisons showing evidence for colocalization (H4) at the main analysis

**Supplementary Table 6.** Results of top 10 SNPs with highest probability in colocalization analysis of GCKR (Ferkingstad et al.) and GDM

**Supplementary Table 7.** Results of top 10 SNPs with highest probability in colocalization analysis of GCKR (Sun et al.) and GDM

**Supplementary Figure 1.** Results of the phenome-wide association study of *cis*-pQTL for GCKR (Ferkingstad et al.)

**Supplementary Figure 2.** Results of the phenome-wide association study of *cis*-pQTL for GCKR (Sun et al.)

**Supplementary Table 1.** The STROBE-MR-checklist applicable to the current study

| **Item No.** | **Section** | **Checklist item** | **Relevant text from manuscript** |
| --- | --- | --- | --- |
| 1 | **TITLE and ABSTRACT** | Indicate Mendelian randomization (MR) as the study’s design in the title and/or the abstract if that is a main purpose of the study | Title; Abstract. |
|  | **INTRODUCTION** |  |  |
| 2 | **Background** | Explain the scientific background and rationale for the reported study. What is the exposure? Is a potential causal relationship between exposure and outcome plausible? Justify why MR is a helpful method to address the study question | Introduction, Para1-3. |
| 3 | **Objectives** | State specific objectives clearly, including pre-specified causal hypotheses (if any). State that MR is a method that, under specific assumptions, intends to estimate causal effects | Introduction, Para3,4. |
|  | **METHODS** |  |  |
| 4 | **Study design and data sources** | Present key elements of the study design early in the article. Consider including a table listing sources of data for all phases of the study. For each data source contributing to the analysis, describe the following: |  |
|  | a) | Setting: Describe the study design and the underlying population, if possible. Describe the setting, locations, and relevant dates, including periods of recruitment, exposure, follow-up, and data collection, when available. | Methods, GWASs involved in current study. |
|  | b) | Participants: Give the eligibility criteria, and the sources and methods of selection of participants. Report the sample size, and whether any power or sample size calculations were carried out prior to the main analysis | Methods, GWASs involved in current study. |
|  | c) | Describe measurement, quality control and selection of genetic variants | Methods, GWASs involved in current study; Genetic instrument selection. |
|  | d) | For each exposure, outcome, and other relevant variables, describe methods of assessment and diagnostic criteria for diseases | Supplementary Table 2. |
|  | e) | Provide details of ethics committee approval and participant informed consent, if relevant | The GWAS summary data used in this study are all publicly available and described in the Supplementary Table 2. |
| 5 | **Assumptions** | Explicitly state the three core IV assumptions for the main analysis (relevance, independence and exclusion restriction) as well assumptions for any additional or sensitivity analysis | Methods, Genetic instrument selection. |
| 6 | **Statistical methods: main analysis** | Describe statistical methods and statistics used |  |
|  | a) | Describe how quantitative variables were handled in the analyses (i.e., scale, units, model) | Supplementary Table 2. |
|  | b) | Describe how genetic variants were handled in the analyses and, if applicable, how their weights were selected | Methods, Genetic instrument selection. |
|  | c) | Describe the MR estimator (e.g. two-stage least squares, Wald ratio) and related statistics. Detail the included covariates and, in case of two-sample MR, whether the same covariate set was used for adjustment in the two samples | Methods, Two-sample MR; Two-step MR for mediation analysis. |
|  | d) | Explain how missing data were addressed | Not applicable. |
|  | e) | If applicable, indicate how multiple testing was addressed | Methods, Two-sample MR. |
| 7 | **Assessment of assumptions** | Describe any methods or prior knowledge used to assess the assumptions or justify their validity | Methods, Two-sample MR; Two-step MR for mediation analysis; Colocalization analysis. |
| 8 | **Sensitivity analyses and additional analyses** | Describe any sensitivity analyses or additional analyses performed (e.g. comparison of effect estimates from different approaches, independent replication, bias analytic techniques, validation of instruments, simulations) | Methods, Two-sample MR; Colocalization analysis. |
| 9 | **Software and pre-registration** |  |  |
|  | a) | Name statistical software and package(s), including version and settings used | Methods, Two-step MR for mediation analysis; Colocalization analysis. |
|  | b) | State whether the study protocol and details were pre-registered (as well as when and where) | Not applicable. |
|  | **RESULTS** |  |  |
| 10 | **Descriptive data** |  |  |
|  | a) | Report the numbers of individuals at each stage of included studies and reasons for exclusion. Consider use of a flow diagram | Supplementary Table 2. |
|  | b) | Report summary statistics for phenotypic exposure(s), outcome(s), and other relevant variables (e.g. means, SDs, proportions) | Supplementary Table 2. |
|  | c) | If the data sources include meta-analyses of previous studies, provide the assessments of heterogeneity across these studies | Not applicable. |
|  | d) | For two-sample MR:  i.  Provide justification of the similarity of the genetic variant-exposure associations between the exposure and outcome samples  ii.  Provide information on the number of individuals who overlap between the exposure and outcome studies | Table 1; Results, Genetic instruments for GCKR. |
| 11 | **Main results** |  |  |
|  | a) | Report the associations between genetic variant and exposure, and between genetic variant and outcome, preferably on an interpretable scale | Results, Genetic instruments for GCKR. |
|  | b) | Report MR estimates of the relationship between exposure and outcome, and the measures of uncertainty from the MR analysis, on an interpretable scale, such as odds ratio or relative risk per SD difference | Results, Association of GCKR with GDM risk and related complications. |
|  | c) | If relevant, consider translating estimates of relative risk into absolute risk for a meaningful time period | Not applicable. |
|  | d) | Consider plots to visualize results (e.g. forest plot, scatterplot of associations between genetic variants and outcome versus between genetic variants and exposure) | Figure 2. |
| 12 | **Assessment of assumptions** |  |  |
|  | a) | Report the assessment of the validity of the assumptions | Results, Association of GCKR with GDM risk and related complications; Mediation MR analysis; Colocalization analysis. |
|  | b) | Report any additional statistics (e.g., assessments of heterogeneity across genetic variants, such as *I^2^*, Q statistic or E-value) | Table 1. |
| 13 | **Sensitivity analyses and additional analyses** |  |  |
|  | a) | Report any sensitivity analyses to assess the robustness of the main results to violations of the assumptions | Results, Mediation MR analysis; Colocalization analysis. |
|  | b) | Report results from other sensitivity analyses or additional analyses | Results, Mediation MR analysis; Colocalization analysis. |
|  | c) | Report any assessment of direction of causal relationship (e.g., bidirectional MR) | Results, Association of GCKR with GDM risk and related complications. |
|  | d) | When relevant, report and compare with estimates from non-MR analyses | Not applicable. |
|  | e) | Consider additional plots to visualize results (e.g., leave-one-out analyses) | None. |
|  | **DISCUSSION** |  |  |
| 14 | **Key results** | Summarize key results with reference to study objectives | Discussion, Para1. |
| 15 | **Limitations** | Discuss limitations of the study, taking into account the validity of the IV assumptions, other sources of potential bias, and imprecision. Discuss both direction and magnitude of any potential bias and any efforts to address them | Discussion, Para7. |
| 16 | **Interpretation** |  |  |
|  | a) | Meaning: Give a cautious overall interpretation of results in the context of their limitations and in comparison with other studies | Discussion, Para2-3. |
|  | b) | Mechanism: Discuss underlying biological mechanisms that could drive a potential causal relationship between the investigated exposure and the outcome, and whether the gene-environment equivalence assumption is reasonable. Use causal language carefully, clarifying that IV estimates may provide causal effects only under certain assumptions | Discussion, Para2-3,. |
|  | c) | Clinical relevance: Discuss whether the results have clinical or public policy relevance, and to what extent they inform effect sizes of possible interventions | Discussion, Para4-6. |
| 17 | **Generalizability** | Discuss the generalizability of the study results (a) to other populations, (b) across other exposure periods/timings, and (c) across other levels of exposure | Discussion, Para8. |
|  | **OTHER INFORMATION** |  |  |
| 18 | **Funding** | Describe sources of funding and the role of funders in the present study and, if applicable, sources of funding for the databases and original study or studies on which the present study is based | Funding statement. |
| 19 | **Data and data sharing** | Provide the data used to perform all analyses or report where and how the data can be accessed, and reference these sources in the article. Provide the statistical code needed to reproduce the results in the article, or report whether the code is publicly accessible and if so, where | Supplementary Table 2. |
| 20 | **Conflicts of Interest** | All authors should declare all potential conflicts of interest | Conflicts of Interest statement. |

**Supplementary Table 2.** Characteristics of the GWAS included in Mendelian randomization analysis

| **Phenotype** | **Description of phenotype** | **GWAS contributing cohorts** | **Sex** | **Covariates** | **Inclusions/exclusions** | **Ancestry** | **Sample size** | **PMID/DOI** |
| --- | --- | --- | --- | --- | --- | --- | --- | --- |
| GCKR | Protein level quantified on a DNA microarray. | The Icelandic Cancer Project, deCODE genetics | Males and females | Age, sex and sample age | Selecte one of the cases of repeated samples for an individual at random. | Icelander | 35,559 | 34857953 |
| GCKR | Using the Affymetrix Axiom UK Biobank genotyping array | INTERVAL study | Males and females | Age, sex, duration between blood draw and processing and the first three principal components (PCs) of ancestry from multi-dimensional scaling. | Individuals were excluded if they a history of major diseases. | European | 3,301 | 29875488 |
| Gestational diabetes mellitus | the International Classification of Diseases (ICD) revisions: ICD-10: O244; ICD-9: 6488A | Finnish biobanks and Finnish health registries | Females | Age, sex, 10 PCs, Finngen 1 or 2 chip or legacy genotyping batch | Sample-wise quality control: individuals with ambiguous gender, high genotype missingness (>5%), excess heterozygosity (+-4SD) and non-Finnish ancestry were excluded.  Variant-wise quality control: variants with high missingness (>2%), low HWE P-value (<1e-6) and minor allele count, MAC<3 were excluded. | European | 9,370 cases, 164,376 controls | <https://doi.org/10.1101/2022.03.03.22271360> |
| Fasting insulin | Fasting insulin measured in pmol/l | MAGIC | Males and females | BMI, study-specific covariates, and principal components (unless implementing a linear mixed model) | Individuals were excluded if they had type 1 or type 2 diabetes (defined by physician diagnosis) | European | 151,013 | 34059833 |
| Fasting glucose |  | MAGIC | Males and females | BMI, study-specific covariates, and principal components (unless implementing a linear mixed model) | Individuals were excluded if they had type 1 or type 2 diabetes (defined by physician diagnosis) | European | 200,622 | 34059833 |
| Body mass index |  | GIANT | Males and Females | Age, sex, recruitment center, genotyping batches and 10 PCs |  | European | 681,275 | 30124842 |
| Triglycerides |  | UK Biobank | Males and Females | Age, sex |  | European | 441,016 | 32203549 |
| Sporadic miscarriage | ICD10: O02.1, O03  Self-reported: 1-2 miscarriages | European-ancestry women | Females | Year of birth | Exclusion: women with early or late menarche (<9 or >17 years); women with any of diagnoses for conditions associated with increased susceptibility to miscarriage (maternal chromosomal abnormalities, thyroid conditions, neoplasms affecting endocrine glands, thrombophilias, disorders affecting the endocrine system, congenital malformations of genital organs) | European | 50,060 cases, 174,109 controls | 33239672 |
| Multiple consecutive miscarriage | ICD10: N96  Self-reported: ≥ 3 miscarriages | European-ancestry women | Females | Year of birth | Exclusion: women with early or late menarche (<9 or >17 years); women with any of diagnoses for conditions associated with increased susceptibility to miscarriage (maternal chromosomal abnormalities, thyroid conditions, neoplasms affecting endocrine glands, thrombophilias, disorders affecting the endocrine system, congenital malformations of genital organs) | European | 750 cases, 150,215 controls | 33239672 |
| Premature rupture of membranes | ICD 10: O42; ICD 9: 6581A; ICD 8: 63495 | Finnish biobanks and Finnish health registries | Females | Age, sex, 10 PCs, Finngen 1 or 2 chip or legacy genotyping batch | Sample-wise quality control: individuals with ambiguous gender, high genotype missingness (>5%), excess heterozygosity (+-4SD) and non-Finnish ancestry were excluded.  Variant-wise quality control: variants with high missingness (>2%), low HWE P-value (<1e-6) and minor allele count, MAC<3 were excluded. | European | 5,066 cases, 142,734 controls | <https://doi.org/10.1101/2022.03.03.22271360> |
| Premature separation of placenta | ICD 10: O45; ICD 9: 6412 | Finnish biobanks and Finnish health registries | Females | Age, sex, 10 PCs, Finngen 1 or 2 chip or legacy genotyping batch | Sample-wise quality control: individuals with ambiguous gender, high genotype missingness (>5%), excess heterozygosity (+-4SD) and non-Finnish ancestry were excluded.  Variant-wise quality control: variants with high missingness (>2%), low HWE P-value (<1e-6) and minor allele count, MAC<3 were excluded. | European | 465 cases, 142,734 controls | <https://doi.org/10.1101/2022.03.03.22271360> |
| Preterm labour and delivery | ICD 10: O60; ICD 9: 644; ICD 8: 63497 | Finnish biobanks and Finnish health registries | Females | Age, sex, 10 PCs, Finngen 1 or 2 chip or legacy genotyping batch | Sample-wise quality control: individuals with ambiguous gender, high genotype missingness (>5%), excess heterozygosity (+-4SD) and non-Finnish ancestry were excluded.  Variant-wise quality control: variants with high missingness (>2%), low HWE P-value (<1e-6) and minor allele count, MAC<3 were excluded. | European | 8,108 cases, 135,806 controls | <https://doi.org/10.1101/2022.03.03.22271360> |
| Eclampsia | ICD 10: O15; ICD 9: 6426 | Finnish biobanks and Finnish health registries | Females | Age, sex, 10 PCs, Finngen 1 or 2 chip or legacy genotyping batch | Sample-wise quality control: individuals with ambiguous gender, high genotype missingness (>5%), excess heterozygosity (+-4SD) and non-Finnish ancestry were excluded.  Variant-wise quality control: variants with high missingness (>2%), low HWE P-value (<1e-6) and minor allele count, MAC<3 were excluded. | European | 385 cases, 160,670 controls | <https://doi.org/10.1101/2022.03.03.22271360> |
| Pre-eclampsia | ICD 10: O14; ICD 9: 642[4-5]; ICD 8: 6370[349] | Finnish biobanks and Finnish health registries | Females | Age, sex, 10 PCs, Finngen 1 or 2 chip or legacy genotyping batch | Sample-wise quality control: individuals with ambiguous gender, high genotype missingness (>5%), excess heterozygosity (+-4SD) and non-Finnish ancestry were excluded.  Variant-wise quality control: variants with high missingness (>2%), low HWE P-value (<1e-6) and minor allele count, MAC<3 were excluded. | European | 5,265 cases, 160,670 controls | <https://doi.org/10.1101/2022.03.03.22271360> |
| Postpartum haemorrhage | ICD 10: O72; ICD 9: 666; ICD 8: 6531 | Finnish biobanks and Finnish health registries | Females | Age, sex, 10 PCs, Finngen 1 or 2 chip or legacy genotyping batch | Sample-wise quality control: individuals with ambiguous gender, high genotype missingness (>5%), excess heterozygosity (+-4SD) and non-Finnish ancestry were excluded.  Variant-wise quality control: variants with high missingness (>2%), low HWE P-value (<1e-6) and minor allele count, MAC<3 were excluded. | European | 6,090 cases, 135,806 controls | <https://doi.org/10.1101/2022.03.03.22271360> |
| Postpartum depression | ICD 10: F32\|F33\|F530 | Finnish biobanks and Finnish health registries | Females | Age, sex, 10 PCs, Finngen 1 or 2 chip or legacy genotyping batch | Sample-wise quality control: individuals with ambiguous gender, high genotype missingness (>5%), excess heterozygosity (+-4SD) and non-Finnish ancestry were excluded.  Variant-wise quality control: variants with high missingness (>2%), low HWE P-value (<1e-6) and minor allele count, MAC<3 were excluded. | European | 11,711 cases, 214,996 controls | <https://doi.org/10.1101/2022.03.03.22271360> |
| Polyhydramnios | ICD 10: O40; ICD 9: 657; ICD 8: 6344 | Finnish biobanks and Finnish health registries | Females | Age, sex, 10 PCs, Finngen 1 or 2 chip or legacy genotyping batch | Sample-wise quality control: individuals with ambiguous gender, high genotype missingness (>5%), excess heterozygosity (+-4SD) and non-Finnish ancestry were excluded.  Variant-wise quality control: variants with high missingness (>2%), low HWE P-value (<1e-6) and minor allele count, MAC<3 were excluded. | European | 903 cases, 142,734 controls | <https://doi.org/10.1101/2022.03.03.22271360> |
| Gestational hypertension | ICD 10: O13; ICD 9: 6423; ICD 8: 63701 | Finnish biobanks and Finnish health registries | Females | Age, sex, 10 PCs, Finngen 1 or 2 chip or legacy genotyping batch | Sample-wise quality control: individuals with ambiguous gender, high genotype missingness (>5%), excess heterozygosity (+-4SD) and non-Finnish ancestry were excluded.  Variant-wise quality control: variants with high missingness (>2%), low HWE P-value (<1e-6) and minor allele count, MAC<3 were excluded. | European | 6,562 cases, 160,670 controls | <https://doi.org/10.1101/2022.03.03.22271360> |
| Primary hypertension | secondary ICD10: I10 Essential (primary) | UK Biobank | Males and Females | Sex, chip and the first 10 PCs |  | European | 54,358 cases, 408,652 controls | 10.5523/bris.pnoat8cxo0u52p6ynfaekeigi |
| Type 2 diabetes | UK Biobank: self-report, ICD10 main diagnoses and ICD10 secondary diagnoses | DIAGRAM, GERA and UK Biobank | Males and Females | GERA: age, sex, and the first 20 PCs  UK Biobank: age, sex |  | European | 62,892 cases, 596,424 controls | 30054458 |

**Supplementary Table 3.** The effect of GCKR instrumented by *cis*-pQTL on GDM related complications

| **Exposure** | **Outcome** | **OR** | **LCI** | **UCI** | **P-value** |
| --- | --- | --- | --- | --- | --- |
| GCKR (Ferkingstad et al.) | Sporadic miscarriage | 1.189 | 1.001 | 1.412 | 0.05 |
|  | Multiple consecutive miscarriage | 1.330 | 0.244 | 7.242 | 0.74 |
|  | Premature rupture of membranes | 0.821 | 0.503 | 1.337 | 0.43 |
|  | Premature separation of placenta | 0.558 | 0.116 | 2.673 | 0.47 |
|  | Preterm labour and delivery | 0.610 | 0.415 | 0.898 | 0.01 |
|  | Eclampsia | 0.463 | 0.083 | 2.592 | 0.38 |
|  | Pre-eclampsia | 0.967 | 0.602 | 1.553 | 0.89 |
|  | Postpartum haemorrhage | 1.453 | 0.931 | 2.267 | 0.10 |
|  | Postpartum depression | 1.039 | 0.742 | 1.457 | 0.82 |
|  | Polyhydramnios | 0.666 | 0.216 | 2.055 | 0.48 |
|  | Gestational hypertension | 1.126 | 0.736 | 1.724 | 0.58 |
|  | Primary hypertension | 0.994 | 0.979 | 1.010 | 0.48 |
|  | Type 2 diabetes | 2.183 | 1.846 | 2.581 | 6.53E-20* |
| GCKR (Sun et al.) | Sporadic miscarriage | 1.119 | 0.978 | 1.280 | 0.10 |
|  | Multiple consecutive miscarriage | 1.263 | 0.333 | 4.783 | 0.73 |
|  | Premature rupture of membranes | 0.873 | 0.596 | 1.277 | 0.48 |
|  | Premature separation of placenta | 0.726 | 0.214 | 2.464 | 0.61 |
|  | Preterm labour and delivery | 0.716 | 0.530 | 0.968 | 0.03 |
|  | Eclampsia | 0.302 | 0.079 | 1.153 | 0.08 |
|  | Pre-eclampsia | 1.001 | 0.692 | 1.448 | 1.00 |
|  | Postpartum haemorrhage | 1.342 | 0.948 | 1.899 | 0.10 |
|  | Postpartum depression | 1.024 | 0.787 | 1.332 | 0.86 |
|  | Polyhydramnios | 0.690 | 0.287 | 1.660 | 0.41 |
|  | Gestational hypertension | 1.081 | 0.776 | 1.506 | 0.65 |
|  | Primary hypertension | 0.994 | 0.982 | 1.006 | 0.32 |
|  | Type 2 diabetes | 1.870 | 1.640 | 2.131 | 8.65E-21* |

Abbreviations: GCKR, glucokinase regulatory protein; GDM, gestational diabetes; OR, odds ratio; LCI, lower confidence interval; UCI, upper confidence interval.

* Significance after Bonferroni correction for multiple testing.

**Supplementary Table 4.** Multivariable Mendelian randomization effect estimates of GCKR on GDM, adjusted for metabolic mediators

| **Exposure** | **Adjusted for** | **Outcome** | **OR** | **LCI** | **UCI** | ***P*-value** |
| --- | --- | --- | --- | --- | --- | --- |
| GCKR | Unadjusted | GDM | 3.466 | 2.401 | 5.002 | 3.16E-11 |
|  | Fasting glucose |  | 1.300 | 0.342 | 4.947 | 7.00E-01 |
|  | Fasting insulin |  | 1.038 | 0.323 | 3.338 | 9.50E-01 |
|  | Triglycerides |  | 2.026 | 1.641 | 2.502 | 5.27E-11 |
|  | BMI |  | 1.019 | 0.814 | 1.276 | 8.71E-01 |

Abbreviations: GCKR, glucokinase regulatory protein; GDM, gestational diabetes; OR, odds ratio; LCI, lower confidence interval; UCI, upper confidence interval.

**Supplementary Table 5.** Results of colocalization analysis for comparisons showing evidence for colocalization (H4) at the main analysis

| **Protein** | **outcome** | **Study** | **PP.H0.abf** | **PP.H1.abf** | **PP.H2.abf** | **PP.H3.abf** | **PP.H4.abf** |
| --- | --- | --- | --- | --- | --- | --- | --- |
| GCKR | GDM | Ferkingstad et al. | 5.74E-26 | 5.53E-21 | 4.05E-08 | 2.91E-03 | 9.97E-01 |
| GCKR | GDM | Sun et al. | 7.02E-08 | 6.62E-03 | 1.20E-07 | 1.03E-02 | 9.83E-01 |

Abbreviations: GCKR, glucokinase regulatory protein; GDM, gestational diabetes; PP, posterior probability.

**Supplementary Table 6.** Results of top 10 SNPs with highest probability in colocalization analysis of GCKR (Ferkingstad et al.) and GDM

| **SNP** | **pvalues.df1** | **MAF.df1** | **N.df1** | **lABF.df1** | **pvalues.df2** | **MAF.df2** | **N.df2** | **lABF.df2** | **Internal.sum.lABF** | **SNP.PP.H4** |
| --- | --- | --- | --- | --- | --- | --- | --- | --- | --- | --- |
| rs1260326 | 3.16E-11 | 0.34132 | 173746 | 19.36084 | 5.75E-25 | 0.34132 | 35373 | 50.15657 | 69.51741 | 0.8935 |
| rs4665972 | 5.83E-11 | 0.33903 | 173746 | 18.76653 | 5.51E-24 | 0.33903 | 35374 | 47.9251 | 66.69162 | 0.052951 |
| rs780094 | 2.84E-11 | 0.33917 | 173746 | 19.46579 | 1.19E-23 | 0.33917 | 35373 | 47.16458 | 66.63037 | 0.049805 |
| rs780093 | 5.67E-11 | 0.34157 | 173746 | 18.79226 | 8.27E-23 | 0.34157 | 35373 | 45.24912 | 64.04139 | 0.00374 |
| rs11127048 | 6.49E-11 | 0.34342 | 173746 | 18.66002 | 6.54E-20 | 0.34342 | 35373 | 38.66535 | 57.32537 | 4.53E-06 |
| rs780095 | 1.83E-08 | 0.41831 | 173746 | 13.16354 | 1.85E-17 | 0.41831 | 35373 | 33.07518 | 46.23871 | 6.94E-11 |
| rs780096 | 1.73E-08 | 0.41895 | 173746 | 13.21762 | 2.10E-17 | 0.41895 | 35373 | 32.95026 | 46.16788 | 6.46E-11 |
| rs6547692 | 1.94E-08 | 0.42103 | 173746 | 13.10645 | 2.36E-17 | 0.42103 | 35373 | 32.83484 | 45.94129 | 5.15E-11 |
| rs2911711 | 2.52E-08 | 0.42008 | 173746 | 12.85426 | 1.44E-16 | 0.42008 | 35373 | 31.0559 | 43.91016 | 6.76E-12 |
| rs1313566 | 2.65E-08 | 0.42008 | 173746 | 12.80572 | 1.40E-16 | 0.42008 | 35373 | 31.08361 | 43.88933 | 6.62E-12 |

Abbreviations: GCKR, glucokinase regulatory protein; GDM, gestational diabetes; SNP, single nucleotide polymorphism; MAF, minor allele frequency; N, study sample size; PP, posterior probability.

**Supplementary Table 7.** Results of top 10 SNPs with highest probability in colocalization analysis of GCKR (Sun et al.) and GDM

| **SNP** | **pvalues.df1** | **MAF.df1** | **N.df1** | **lABF.df1** | **pvalues.df2** | **MAF.df2** | **N.df2** | **lABF.df2** | **internal.sum.lABF** | **SNP.PP.H4** |
| --- | --- | --- | --- | --- | --- | --- | --- | --- | --- | --- |
| rs780094 | 2.84E-11 | 0.39249 | 173746 | 19.44329 | 1.15E-05 | 0.39249 | 3301 | 7.560503 | 27.00379 | 0.381457 |
| rs1260326 | 3.16E-11 | 0.40151 | 173746 | 19.33654 | 1.91E-05 | 0.40151 | 3301 | 7.088135 | 26.42467 | 0.213766 |
| rs11127048 | 6.49E-11 | 0.39285 | 173746 | 18.63929 | 1.23E-05 | 0.39285 | 3301 | 7.498003 | 26.13729 | 0.160372 |
| rs780093 | 5.67E-11 | 0.3924 | 173746 | 18.77078 | 1.51E-05 | 0.3924 | 3301 | 7.307832 | 26.07861 | 0.151232 |
| rs4665972 | 5.83E-11 | 0.39883 | 173746 | 18.74163 | 2.51E-05 | 0.39883 | 3301 | 6.835754 | 25.57738 | 0.091614 |
| rs6547692 | 1.94E-08 | 0.44975 | 173746 | 13.10029 | 5.37E-05 | 0.44975 | 3301 | 6.12476 | 19.22505 | 0.00016 |
| rs780095 | 1.83E-08 | 0.43965 | 173746 | 13.1585 | 5.89E-05 | 0.43965 | 3301 | 6.040784 | 19.19928 | 0.000156 |
| rs2068834 | 2.89E-08 | 0.26524 | 173746 | 12.81098 | 6.03E-05 | 0.26524 | 3301 | 6.078173 | 18.88916 | 0.000114 |
| rs4666000 | 2.87E-08 | 0.26721 | 173746 | 12.81585 | 7.59E-05 | 0.26721 | 3301 | 5.866636 | 18.68248 | 9.28E-05 |
| rs780096 | 1.73E-08 | 0.44168 | 173746 | 13.21236 | 0.00011 | 0.44168 | 3301 | 5.468707 | 18.68106 | 9.27E-05 |

Abbreviations: GCKR, glucokinase regulatory protein; GDM, gestational diabetes; SNP, single nucleotide polymorphism; MAF, minor allele frequency; N, study sample size; PP, posterior probability.

**Supplementary Figure 1.** Results of the phenome-wide association study of *cis*-pQTL for GCKR (Ferkingstad et al.)


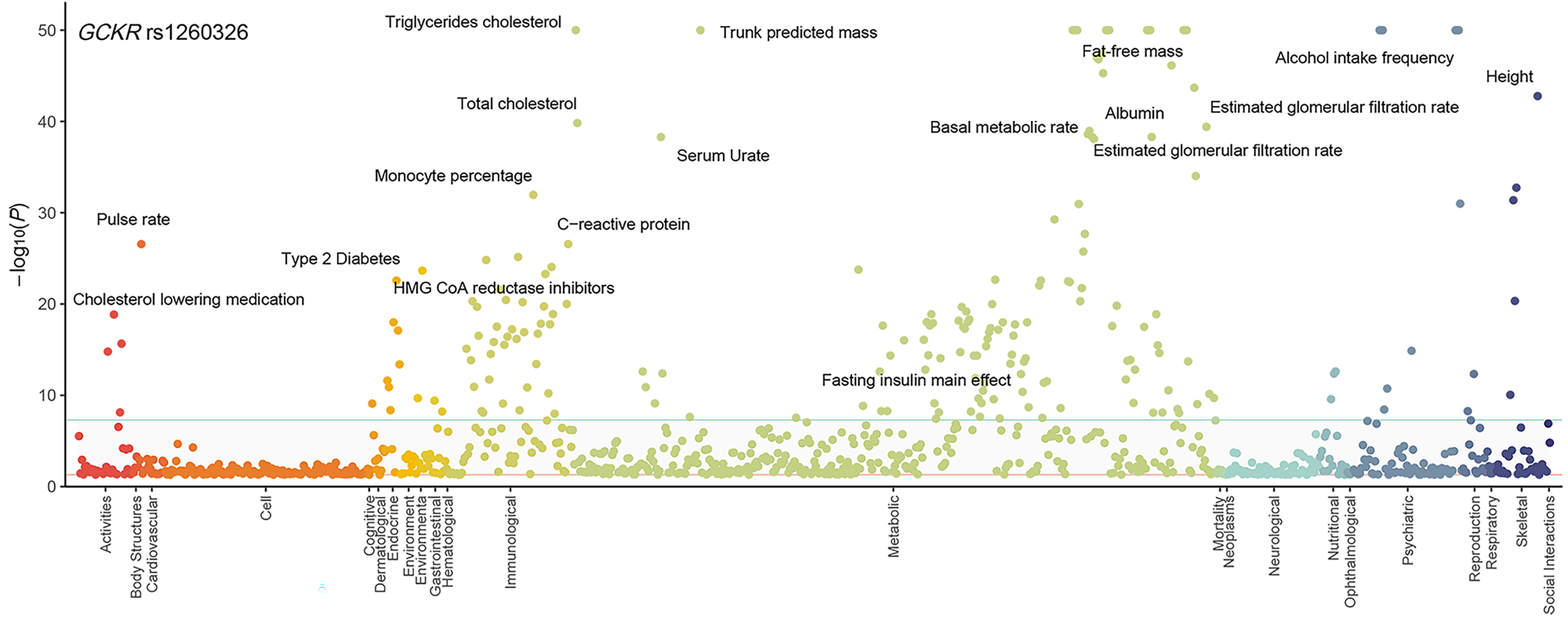


The traits with P-value less than 1.0×10^-50^ were annotated as 1.0×10^-50^ in the figure.

**Supplementary Figure 2.** Results of the phenome-wide association study of *cis*-pQTL for GCKR (Sun et al.)


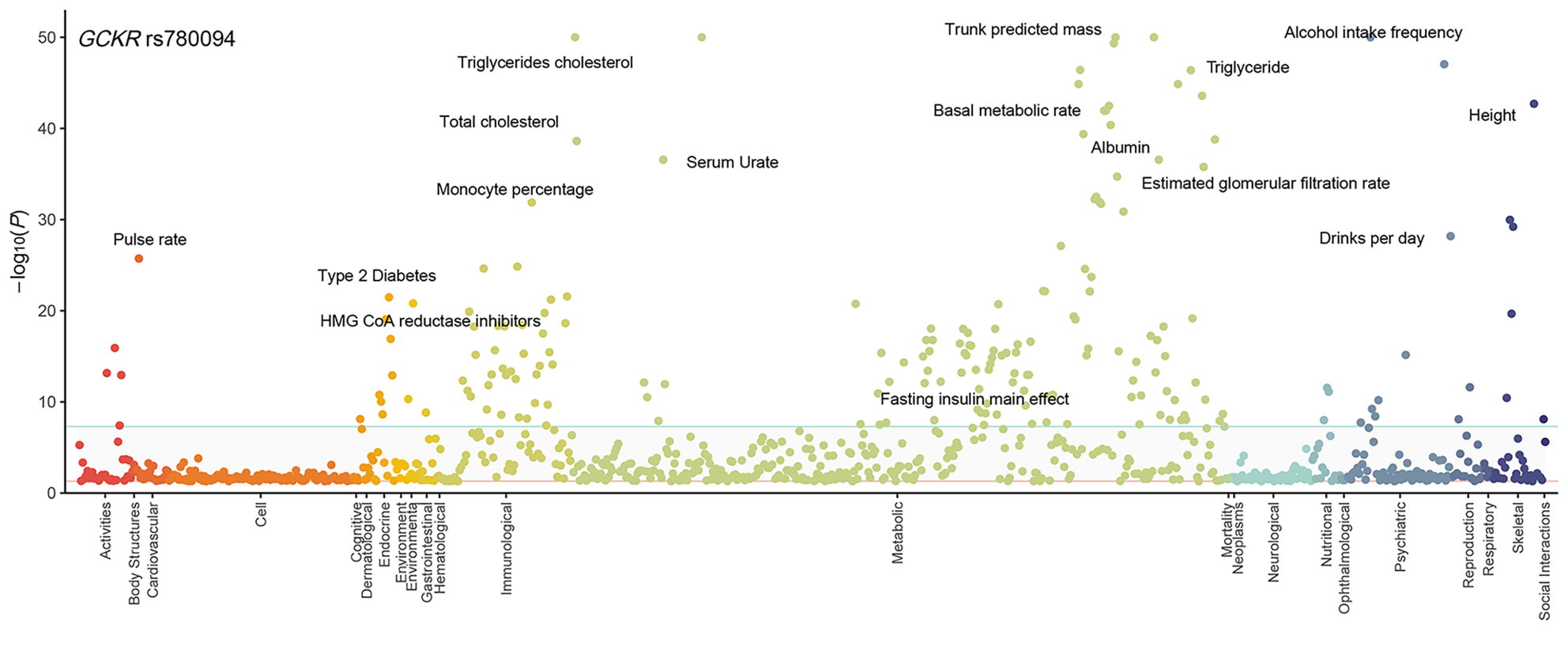


The traits with P-value less than 1.0×10^-50^ were annotated as 1.0×10^-50^ in the figure.
